# RVINN: a flexible modeling for inferring dynamic transcriptional and post-transcriptional regulation using physics-informed neural networks

**DOI:** 10.1093/bioinformatics/btaf180

**Published:** 2025-07-15

**Authors:** Osamu Muto, Zhongliang Guo, Rui Yamaguchi

**Affiliations:** Division of Cancer Systems Biology, Aichi Cancer Center Research Institute, Nagoya, 464-8681, Japan; Division of Cancer Informatics, Nagoya University Graduate School of Medicine, Nagoya, 466-8550, Japan; Division of Cancer Systems Biology, Aichi Cancer Center Research Institute, Nagoya, 464-8681, Japan; Division of Cancer Informatics, Nagoya University Graduate School of Medicine, Nagoya, 466-8550, Japan; Division of Cancer Systems Biology, Aichi Cancer Center Research Institute, Nagoya, 464-8681, Japan; Division of Cancer Informatics, Nagoya University Graduate School of Medicine, Nagoya, 466-8550, Japan

## Abstract

**Summary:**

Dynamic gene expression is controlled by transcriptional and post-transcriptional regulation. Recent studies on transcriptional bursting and buffering have increasingly highlighted the dynamic gene regulatory mechanisms. However, direct measurement techniques still face various constraints and require complementary methodologies, which are both comprehensive and versatile. To address this issue, inference approaches based on transcriptome data and differential equation models representing the messenger RNA lifecycle have been proposed. However, the inference of complex dynamics under diverse experimental conditions and biological scenarios remains challenging. In this study, we developed a flexible modeling using physics-informed neural networks and demonstrated its performance using simulation and experimental data. Our model has the ability to computationally revalidate and visualize dynamic biological phenomena, such as transcriptional ripple, co-bursting, and buffering in a breast cancer cell line. Furthermore, our results suggest putative molecular mechanisms underlying these phenomena. We propose a novel approach for inferring transcriptional and post-transcriptional regulation and expect to offer valuable insights for experimental and systems biology.

**Availability and implementation:**

https://github.com/omuto/RVINN.

## 1 Introduction

The temporal dynamics of gene expression in cellular systems are observed in various patterns under different biological contexts and conditions (de Nadal *et al.* 2011). For example, oscillating gene expression patterns are represented by circadian genes and dynamic steady-state transitions or stable gene expression patterns that occur under extracellular perturbations such as growth factors or drugs. Deciphering the temporal regulation of gene expression is the key to understanding the fundamental biology and pathological cellular systems of diseases such as cancer.

This study focused on the gene regulatory mechanisms that control the messenger RNA (mRNA) lifecycle. The regulatory steps of the mRNA lifecycle can be classified into two main processes: transcriptional and post-transcriptional regulation. The former process corresponds to pre-mRNA transcription, whereas the latter includes splicing and mRNA degradation. Recent studies focusing on transcriptional bursting and buffering have highlighted the importance of dynamic changes in the regulation of transcription and degradation (Timmers and Tora 2018, Tunnacliffe and Chubb 2020). These findings were demonstrated through specialized experiments using live imaging or metabolic labeling techniques (Sun *et al.* 2012, Hoppe and Ashe, 2021). Nevertheless, it remains challenging to observe both transcriptional and post-transcriptional effects on individual genes simultaneously in a temporal and genome-wide manner for the following reasons: First, the live imaging of transcription dynamics requires the insertion of synthetic probe sequences into specific transcribed DNA regions (Hoppe and Ashe 2021). Thus, we can observe only a limited number of genes that were selected in advance. Next, metabolic labeling methods utilizing nucleoside analogs such as 4-thiouridine (4sU) have issues related to time resolution, cytotoxic effects, and their potential impacts on splicing efficiency (Russo *et al.* 2017, Altieri and Hertel 2021, Moreno *et al.* 2022). They are based on the incorporation of 4sU into synthesizing RNA molecules after adding it to cell culture media and have been used to estimate the amount of nascent RNA and its half-life. However, this approach tends to complicate experimental procedures, and the time resolution of the transcription rate depends on the labeling duration and sampling interval. Regarding the estimation of RNA half-life, it is challenging to address temporal changes in the RNA half-life during steady-state transitions associated with biological perturbations (Russo *et al.* 2017). In addition, there are concerns regarding experimental artifacts when using nucleoside analogs (Altieri and Hertel 2021, Moreno *et al.* 2022). They could alter global gene expression levels caused by the experimental procedures themselves and might be unsuitable for studies focusing on cellular responses after drug perturbations or similar conditions.

In this context, there is a growing demand for tools to infer the global and temporal dynamics of transcriptional and post-transcriptional regulation without specialized experiments. Therefore, a framework combining conventional transcriptomic data with an ordinary differential equation (ODE) model of the mRNA lifecycle has been proposed (Zeisel *et al.* 2011, La Manno *et al.* 2018, Furlan *et al.* 2020). This framework leverages unspliced (intronic) pre-mRNA signals or sequencing reads as surrogates for nascent mRNA (Gaidatzis *et al.* 2015, Wu *et al.* 2022), and its ODE model represents the dynamics of transcriptional and post-transcriptional regulation with the kinetic parameters of transcription, splicing, and mRNA degradation. Although the framework, including the ODE model, was originally proposed on the basis of the bulk time-course microarray data in cultured cell lines (Zeisel *et al.* 2011), it has also been applied to time-course bulk RNA-sequencing (RNA-seq) and single-cell RNA-seq data (La Manno *et al.* 2018, Furlan *et al.* 2020). In particular, single-cell RNA-seq analysis using the framework is known as “RNA velocity analysis” to infer temporal direction of cellular state transitions by estimating each kinetic parameter (La Manno *et al.* 2018, Bergen *et al.* 2020). Its utility has been evaluated using diverse datasets, and multiple methods based on this model have been developed (Bastidas-Ponce *et al.* 2019, Zheng *et al.* 2021, Lange *et al.* 2022, Gayoso *et al.* 2024).

However, it remains tricky to estimate each kinetic parameter required to satisfy the system of the ODE model using observed data (see also Existing Approaches in Methods section). Analytical approaches typically require explicit functional forms for the temporal dynamics of the observed data and/or kinetic parameters (Zeisel *et al.* 2011, Furlan *et al.* 2020). Consequently, these analytical approaches may be limited in their ability to capture a wide variety of biological and temporal dynamics. Therefore, we drew inspiration from physics-informed neural networks (PINNs) to address this challenge. A PINN is a machine learning method that leverages the function approximation capabilities of neural networks and automatic differentiation to incorporate prior knowledge of the underlying data into a model in the form of differential equations and/or derivative values (Raissi *et al.* 2019).

In this study, we introduce RNA velocity-informed neural networks (RVINNs), a new method that enables the flexible modeling of transcriptional and post-transcriptional regulatory dynamics. By modeling each gene independently, RVINN aims to estimate gene-specific and interpretable dynamics of kinetic parameters and provide biological insights and hypotheses. First, we applied RVINN to the simulated data to evaluate its ability to recover the ground-truth dynamics. We then applied this method to publicly available data from the MCF-7 breast cancer cell line that had been treated with different extracellular perturbations. We demonstrated its utility in genome-wide analyses of dynamic biological phenomena, such as the transcriptional ripple effect and co-bursting, both of which are linked to genomic distances and enhancer elements (Ebisuya *et al.* 2008, Kawasaki and Fukaya 2023). Finally, we spotlighted the dynamic aspects of transcriptional buffering, an RNA homeostasis mechanism that balances RNA transcription and degradation following perturbations and discussed evidence suggesting that RNA-binding proteins may be involved in this dynamic process.

## 2 Materials and methods

### 2.1 ODE model of mRNA lifecycle

**Figure 1.**
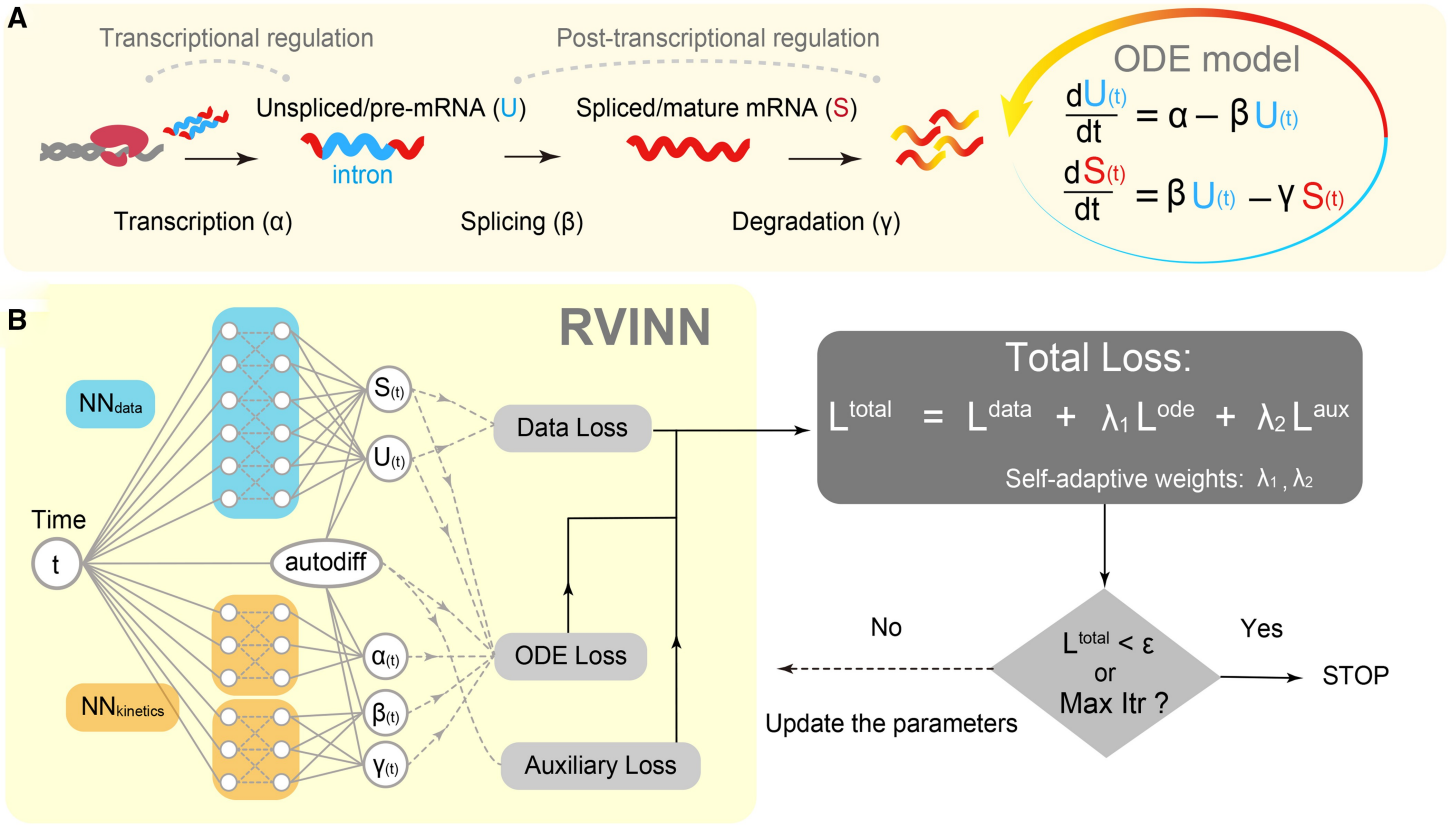
Overview of the ODE model of the mRNA lifecycle (A) and the RVINN framework (B). In the ODE model, the dynamics of unspliced mRNA (U) are governed by the transcription rate (α) and splicing rate (β), while spliced mRNA (S) is modeled by the splicing rate (β) and degradation rate (γ). The RVINN framework models time-course gene expression data using neural networks, leveraging automatic differentiation (autodiff) to compute the ODE and Auxiliary loss.

Following the mathematical formulation and notation by Zeisel *et al.* (2011) and La Manno *et al.* (2018), we use the mRNA lifecycle model based on two coupled ODEs that describe the two molecular states of mRNA, U(t) and S(t) for each gene, representing the abundance of unspliced and spliced mRNA over continuous time $t\in R_{\geq 0}$, respectively (Fig. 1A). Each kinetic parameter in these ODEs corresponds to the transcription rate α, splicing rate β, and degradation rate γ:


$$\begin{array}{c} \frac{dU(t)}{dt}=\alpha -\beta U(t),\\ \frac{dS(t)}{dt}=\beta U(t)-\gamma S(t). \end{array}$$

Suppose that each kinetic parameter can be represented as a time-dependent scalar function, i.e. $\alpha (t),\beta (t),\gamma (t):R_{\geq 0}\to R_{>0}$. Then, we rewrote the system of the above ODEs as follows:


(1) $$\frac{dy(t)}{dt}=F{\left(y(t),\alpha (t),\beta (t),\gamma (t)\right)}_{,}$$

where


(2) $$\frac{dy(t)}{dt}=\frac{d}{dt}\left(\begin{array}{c} U(t)\\ S(t) \end{array}\right),$$

(3) $$\begin{array}{c} F(y(t),\alpha (t),\beta (t),\gamma (t))=\left(\begin{array}{ccc} \alpha (t) & -\beta (t) & 0\\ 0 & \beta (t) & -\gamma (t) \end{array}\right){\left(\begin{array}{c} 1\\ U(t)\\ S(t) \end{array}\right)}_{.} \end{array}$$

### 2.2 Existing approaches

Our aim was to estimate unknown kinetic parameters and mRNA levels over continuous time, including unobserved intervals, while satisfying the nonlinear dynamic system described in Equation (1). As reviewed in Furlan *et al.* (2021), several methods have been proposed to estimate transcriptional and post-transcriptional rates in a snapshot under steady-state conditions. In contrast to methods based on steady-state, to the best of our knowledge, only two approaches by Zeisel *et al.* (2011) and INSPEcT (Furlan *et al.* 2020) have been developed to estimate temporal kinetic parameters based on a formulation equivalent to Equation (1) and standard time-course bulk transcriptomic data without requiring nascent RNA measurements. However, analytically solving Equation (1) becomes intractable unless specific functional forms are assumed for the unobserved dynamics of mRNA levels and/or kinetic parameters. Thus, both of the existing approaches rely on applying tractable functional forms to solve the equation.

In case of Zeisel *et al.* (2011), they demonstrated that splicing rate remains time-invariant under perturbation conditions through transcription inhibition experiments. Using experimentally determined time-invariant values of splicing rate for each gene reduces the number of time-varying parameters, allowing them to focus on transcription and degradation rates. However, this approach still confines the mRNA dynamics to a simple five-parameter model, thereby restricting the range of possible biological behaviors like oscillating behavior. In addition, transcription inhibition experiments are necessary in this approach and its publicly available implementation has not been provided.

INSPEcT (Furlan *et al.* 2020), the only comparable method in this study, addresses this problem by offering two computational modes: the nonfunctional (NF) mode and the default mode. In both modes, the analysis starts by modeling the observed data as piecewise functions, enabling an analytical approach. Transcription rates are modeled as piecewise linear functions, whereas post-transcriptional parameters are modeled as piecewise constant functions, providing the first estimate for each observation interval. In the NF mode, the estimated parameters remain as piecewise functions. By contrast, the default mode offers three functional forms (constant, sigmoid, and impulse). The model selects the most appropriate form for each parameter, guided by an information criterion that balances the data fitness and model complexity. Although INSPEcT supports time-varying parameter estimation, it has reliance on piecewise functions often complicates the smooth representation of the state and parameter trajectories, particularly when the observation intervals are large.

To address this challenge, we propose an approach inspired by PINN (Yazdani *et al.* 2020, Kharazmi *et al.* 2021). In solving inverse problems with a PINN-based model, the goal is to estimate the true kinetic parameters within the observed temporal domain by fitting neural networks (NNs) to observed data under the regularizing guidance of structured differential equations with unknown kinetic parameters. We propose a new method, RVINN, and applied it to estimate the dynamics of gene expression and kinetic parameters for thousands of genes by employing a loss function that embeds the ODE model as prior knowledge.

### 2.3 RVINN design

RVINN consists of two main NN modules for the data and kinetic parameters, both of which require time as input. The data module fits time-course gene expression data (Fig. 1B, blue module) to minimize the data loss. The kinetic modules estimate the dynamics of each transcriptional and post-transcriptional parameter (Fig. 1B, orange modules). Both modules are linked through a loss term of the ODE residuals based on Equation (1) by calculating the time derivatives of each mRNA state using automatic differentiation. Additionally, we included a soft constraint on the time derivative of the splicing rate β(t) as an auxiliary loss in RVINN based on the assumption that splicing efficiency does not drastically change over short timescales in a gene-specific manner. Although RVINN can run without this constraint, we included it to naturally incorporate prior knowledge about the relative lack of gene-specific molecular mechanisms that can dynamically regulate the efficiency of the splicing step compared to the transcription and degradation steps in this study. To simply explain the RVINN loss functions, let us consider a time-course dataset without replicates ${\left\{t_{i},Y_{i}\right\}}_{i=1}^{N^{\mathrm{data}}}$ which consists of $N^{\mathrm{data}}$ pairs of time points ${\left\{t_{i}\right\}}_{i=1}^{N^{\mathrm{data}}}$ as inputs and measured data ${\left\{Y_{i}\right\}}_{i=1}^{N^{\mathrm{data}}}$ as target outputs over the observation period [0,T]. We also consider $N^{\mathrm{ode}}$ arbitrary time points ${\left\{t_{c}\right\}}_{c=1}^{N^{\mathrm{ode}}}$ as inputs with arbitrary time intervals to evaluate the ODE residuals, where $t_{c}\in [0,T]$ and $N^{\mathrm{ode}}\gg N^{\mathrm{data}}$. The total loss function of RVINN is defined as follows:


(4) $$L_{\mathrm{total}}(\theta ,\lambda_{1},\lambda_{2})=L_{\mathrm{data}}(\theta )+\lambda_{1}L_{\mathrm{ode}}(\theta )+\lambda_{2}L_{\mathrm{aux}}(\theta ),$$

where


(5) $$L_{\mathrm{data}}(\theta )=\frac{1}{N^{\mathrm{data}}}\sum_{i=1}^{N^{\mathrm{data}}}{\left[\;Y(t_{i})-y_{\theta }(t_{i})\right]}_{,}^{2}$$

(6) $$L_{\mathrm{ode}}(\theta )=\frac{1}{N^{\mathrm{ode}}}\sum_{c=1}^{N^{\mathrm{ode}}}{\left[{\frac{dy_{\theta }}{dt}|}_{t_{c}}-F(y_{\theta }(t_{c}),\alpha_{\theta }(t_{c}),\beta_{\theta }(t_{c}),\gamma_{\theta }(t_{c}))\right]}_{,}^{2}$$

(7) $$L_{\mathrm{aux}}(\theta )=\frac{1}{N^{\mathrm{ode}}}\sum_{c=1}^{N^{\mathrm{ode}}}{\left|{\frac{d\beta_{\theta }}{dt}|}_{t_{c}}\right|}_{.}$$

Here, $y_{\theta }(\cdot )$, $\alpha_{\theta }(\cdot )$, $\beta_{\theta }(\cdot )$, and $\gamma_{\theta }(\cdot )$ are the values predicted by NN modules, and θ represent the set of NN weights. By default, the data module has two hidden layers, each with 2×  $N^{\mathrm{data}}$ nodes using tan*h* activation function. The kinetic modules use the same architecture, except that their node number is set to a maximum of 2×  $N^{\mathrm{data}}$ or 32. $\lambda_{1}$ and $\lambda_{2}$ are the hyperparameters that control the regularization strength. In this study, self-adaptive weighting is applied to these parameters during the training step (McClenny and Braga-Neto 2023). This technique is commonly used to stabilize the training of PINN in both forward and inverse problems, and is implemented via the following min–max optimization:


(8) $$\underset{\theta }{\min}\;\;\underset{\lambda_{1,2}}{\max}\;L_{\mathrm{total}}(\theta ,\lambda_{1},\lambda_{2}).$$

Concretely, each λ is initialized with a small value (e.g. 0.01 or $\frac{1}{N^{\mathrm{data}}}$) and is gradually increased by optimizers such as Adam and L-BFGS using gradient ascent, as long as the total loss continues to decrease. In essence, the early training steps prioritize fitting to the general patterns of the observed data, whereas the later steps shift more focus to the unobserved states of the data and on the estimation of kinetic parameters. Training continues for up to 3000 iterations with Adam and up to 5000 iterations with L-BFGS or until the total loss converges. Since the training results exhibited a certain degree of variation depending on the random initialization of NN, we usually performed 10 training sets for each gene and used the averaged estimated dynamics for downstream analyses.

In addition to the default loss functions described above, the following optional loss terms can be used for the initial states at $t_{1}$ (t=0):


(9) $$L_{\mathrm{data}}^{\mathrm{init}}(\theta )=L_{\mathrm{data}}(\theta )+{\left[\;Y(0)-y_{\theta }(0)\right]}^{2}$$

and


(10) $$L_{\mathrm{ode}}^{\mathrm{steady}}(\theta )=L_{\mathrm{ode}}(\theta )+{\left[F(y_{\theta }(0),\alpha_{\theta }(0),\beta_{\theta }(0),\gamma_{\theta }(0))\right]}_{.}^{2}$$

A common biological experimental design involves perturbing cells with specific agents and measuring gene expression over time. In such scenarios, we empirically observed that RVINN sometimes struggles to capture drastic changes in the measured data during the early transition from the initial steady-state, causing the initial values to be overlooked. To mitigate this issue, we provide the optional loss functions detailed in Equations (9) and (10). In this article, we employ the default total loss in Equation (4) unless stated otherwise.

### 2.4 Data preparation

#### 2.4.1 Simulation data

To evaluate the baseline performance of RVINN in estimating kinetic parameters, we generated simulated datasets of time-course gene expression using the ODE model with an ODE solver (scipy.integrate.odeint) and kinetic parameter dynamics (see also the Supplementary Materials Section S1). We considered the following biological scenarios for the transcription rate α(t) and the degradation rate γ(t):

1. Steady-to-steady scenario: This scenario simulates how cells experience transient or sustained transcriptional and post-transcriptional regulation, eventually returning to their initial steady states or transitioning to different steady states under perturbations or cellular stress.
2. Oscillating scenario: This scenario simulates oscillating gene expression patterns arising from periodic transcriptional and post-transcriptional regulation.

We generated two datasets based on these scenarios. From each scenario, we evaluated 1000 genes that were weighted-sampled to match the coefficient-of-variation distribution of the simulated data with that of the real data. We performed numerical simulations over a 24-h period (1440 min) and sampled data at three intervals: 25 time points (sampling interval: SI = 60 min), 13 time points (SI = 120 min), and nine time points (SI = 180 min). For each sampling interval, we generated triplicates by adding Gaussian noise to the simulated trajectory at levels corresponding to 10%, 20%, and 30% of the standard deviation of the trajectory. Each model was trained for each gene using sampled data from the observed time period (0–1440 min), and the estimated parameter dynamics were evaluated against the ground-truth dynamics over the same period using the cross-correlation coefficient (lag 0) and the relative $L_{1}$-error.

#### 2.4.2 Time-course bulk RNA-seq data

Raw data were obtained from Baran-Gale *et al.* (2016) and Diaz *et al.* (2020) via NCBI GEO. Both datasets included time-course RNA-seq experiments performed on MCF-7 breast cancer cell line. The dataset from Baran-Gale *et al.* measured the MCF-7 transcriptome at 10 time points (0, 60, 120, 180, 240, 300, 360, 480, 720, and 1440 min), each in triplicate, after treatment with 10 nM estradiol (E2). The dataset from Diaz *et al.* measured the MCF-7 transcriptome at six time points (0, 180, 360, 540, 720, and 1440 min), each in triplicate, following treatment with 20 μM tamoxifen (TAM). Raw FASTQ files were preprocessed using fastp (ver 0.23.4) (Chen *et al.* 2018b), and the sequencing reads were subsequently mapped to the GRCh38 human genome reference using STAR (ver 2.6.1c for the E2 dataset, ver 2.7.11a for the TAM dataset) (Dobin *et al.* 2013). The intronic and exonic read counts for each gene were counted using TPMCalculator (ver 0.0.4) (Vera Alvarez *et al.* 2019) and converted to counts per million (CPM) levels based on each sample’s library size. We then normalized the intronic and exonic CPM levels for each gene using their respective maximum values across all time points, ensuring that the normalized values were dimensionless and capped at 1. We used these normalized values as proxies for pre-mRNA and mature mRNA levels and trained RVINN with the optional loss functions based on Equations (9) and (10). For the large-scale dataset described above, all training runs were executed on a high-performance computing (HPC) cluster using array jobs and CPU resources.

## 3 Results

The experiments described in the Results section consist of two main parts: (1) evaluation of RVINN’s performance in estimating kinetic parameters using simulation data, and (2) investigation of genome-wide dynamic gene regulation and potential molecular mechanisms using real data from a breast cancer cell line. Details are presented in the following subsections.

### 3.1 Simulation-based evaluation of RVINN

**Figure 2.**
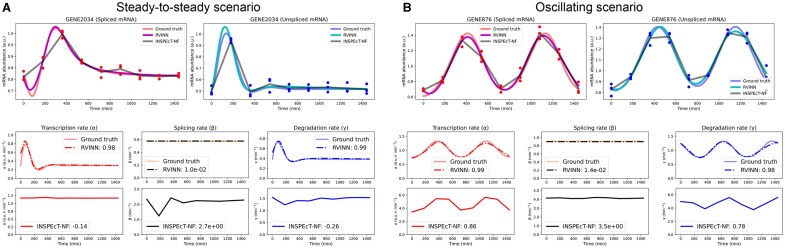
Examples of time-course gene expression data simulated under the steady-to-steady scenario (A) and the oscillating scenario (B). Each dot represents an observed data (red for mature mRNA, blue for pre-mRNA), sampled at nine time points in triplicate with 30% noise. The *x*-axis is time (min), and the *y*-axis is mRNA abundance. a.u. indicates arbitrary units. Upper panels in (A) and (B) show time-course profiles from the trained data modules of RVINN (solid magenta or cyan lines) and INSPEcT (NF mode) with linear interpolation (solid gray lines). Lower panels in (A) and (B) show the time-course profiles of the estimated kinetic parameters by RVINN (dashed lines in color) and INSPEcT (NF mode; solid lines in color), compared with the ground truth. Legends for α and γ show the cross-correlation coefficient (lag 0), whereas the legend for β indicates the relative $L_{1}$-error.

We first evaluated the performance of RVINN using the simulated datasets and compared it with that of INSPEcT (NF mode). RVINN performed well in estimating kinetic parameters with median cross-correlation coefficients ranging from 0.93 to 0.97 for the transcription rate, 0.87 to 0.94 for the degradation rate, and relative $L_{1}$-errors for the splicing rate ranging from $1.9\times 10^{-1}$ to $4.8\times 10^{-1}$ across all datasets (Supplementary Fig. S3 and Table S1). In comparison, INSPEcT (NF mode) showed lower performance with median cross-correlation coefficients ranging from 0.31 to 0.89 for the transcription rate, 0.44 to 0.81 for the degradation rate, and relative $L_{1}$-errors for the splicing rate ranging from 2.3 to 3.9. Furthermore, RVINN showed lower variability in its evaluation metrics than the other method, indicating a robust performance across different functional forms and experimental setups. We also evaluated the other mode of INSPEcT (default mode), which models kinetic parameters using constant, sigmoid, and impulse functions, but observed similar results (Supplementary Table S2). It should be noted that the underlying principles and assumptions differ among the methods, and a direct comparison of their performances is not straightforward. However, RVINN enables smooth data representation throughout the entire observation period, which particularly highlights the differences from analytical methods that utilize piecewise functions for data or parameter representation (Fig. 2A and B). We also attempted to reconstruct smooth gene expression profiles using the kinetic parameters and initial values estimated by INSPEcT with an ODE solver. However, we still observed a tendency for sharp bends near the observation points, possibly due to the use of piecewise functions (Supplementary Fig. S4). These results suggest that RVINN not only recovers unobserved dynamics in simulated scenarios, but also provides a flexible framework for modeling complex and non-linear gene regulation.

### 3.2 Exploring transcriptional ripple effects and enhancer-driven regulation

**Figure 3.**
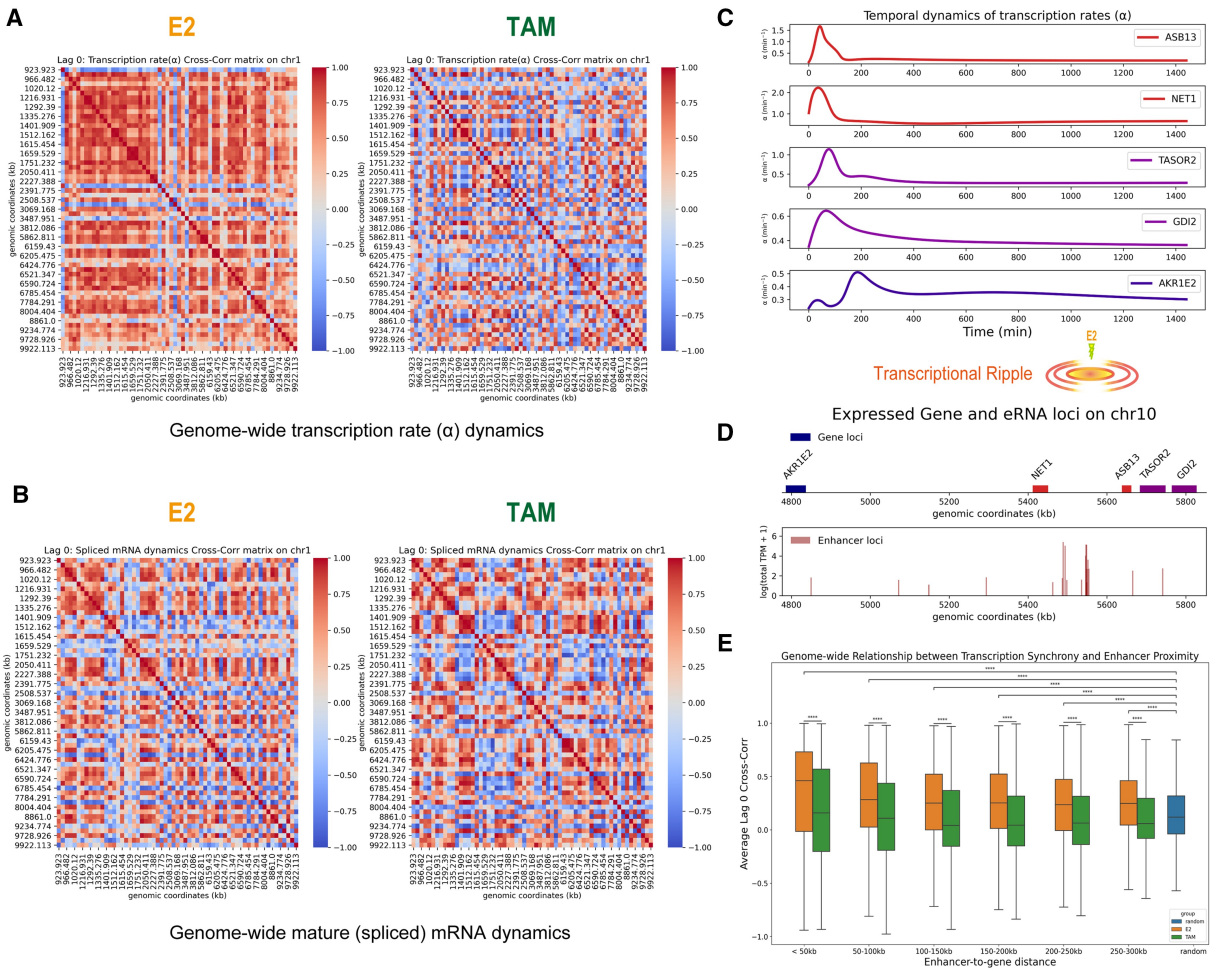
Genome-wide analysis of transcription dynamics under estradiol (E2) and tamoxifen (TAM) treatments. Cross-correlation matrices of transcription rate (A) and spliced mRNA (B) dynamics for genes on chromosome 1 (0–10 000 kb) under E2 (left) and TAM (right) treatments. Each symmetric matrix is indexed by gene start positions and represents the lag-0 cross-correlation between every pair of genes. Warmer colors indicate higher correlation. kb: kilobases. (C) Temporal delay in transcription rates on a genomic region containing expressed genes ASB13, NET1, TASOR2, GDI2, and AKR1E2 under E2 treatment. The plots highlight gene-specific differences in transcriptional activation over time. (D) Genomic coordinates of the expressed genes under E2 treatment and the MCF-7 enhancer RNA (eRNA) loci on chromosome 10. Red peaks in the bottom panel indicate eRNA expression in transcripts per million (TPM) obtained from FANTOM5 (Andersson *et al.* 2014). (E) Genome-wide analysis of transcriptional synchronicity and enhancer proximity. Box plots show average lag-0 cross-correlation coefficients for genes grouped by 50 kb binned genomic distance from the active enhancer regions. The active enhancer regions were defined by eRNA loci with log(total TPM + 1) >4.0. The box plots in orange, green, and blue represent the E2 treatment, TAM treatment, and randomly selected genes from outside the active enhancer regions in the E2 dataset, respectively. One-sided Mann–Whitney *U*-tests were performed on the average correlation coefficients. Asterisks indicate significant pairs based on adjusted *P* < .05 by the Benjamini–Hochberg (BH) method (****: adjusted *P* $\leq 1.0\times 10^{-4}$). Pairs with no annotations were not tested.

We applied RVINN to real-world datasets to infer genome-wide and dynamic gene regulation using estimated kinetic parameters. In this section, we focus on the time-course transcription rate profiles of 7866 coding genes co-expressed in MCF-7 cells under both E2 and TAM treatments. MCF-7 is an estrogen receptor (ER)-positive breast cancer cell line that is well known for its dynamic gene expression changes in response to E2 treatment (Comşa *et al.* 2015). TAM is an ER antagonist that is commonly used to treat ER-positive breast cancer (Clarke *et al.* 2003). As Saravanan *et al.* (2020) have reported ligand-dependent ER clusters formation across the genome in MCF-7 using live imaging, we attempted to investigate the genome-wide similarity of temporal transcription patterns along genomic coordinates as a first step. Interestingly, we found a marked difference of temporal correlation patterns between E2 and TAM treatments (Fig. 3A). This result may reflect the difference of the efficacy in ER-clusters formulation between E2 and TAM. We also found that this difference of correlation patterns between E2 and TAM treatments was not relatively clear in spliced (mature) mRNA dynamics (Fig. 3B), suggesting that RVINN provides a novel perspective beyond conventional time-course gene expression analyses.

Next, we explored the transcriptional ripple effect, a dynamic phenomenon in which the transcriptional activation of highly upregulated genes influences the neighboring genomic regions (Ebisuya *et al.* 2008). As shown in Fig. 3C and Supplementary Fig. S5, we observed intriguing patterns of transcriptional delay and spread over time within a genomic region containing the known E2-responsive gene ASB13 (Liberzon *et al.* 2015). In addition, we observed a potential link between delayed transcriptional activation and enhancer RNA (eRNA) expression (Fig. 3D). As previous studies based on live imaging have reported that the transcriptional co-bursting is regulated by enhancers (Kawasaki and Fukaya 2023), we performed a genome-wide analysis of the average lag-0 cross-correlation coefficients in gene groups defined by enhancer proximity (Fig. 3E). As expected, this analysis showed that gene groups closer to enhancer regions tended to exhibit temporally synchronized transcriptional patterns, and that this synchronization diminished as the enhancer-to-gene distance increased. These results suggest that RVINN is an insightful tool for studying transcriptional regulation and dynamics, particularly on a genome-wide scale.

### 3.3 RVINN spotlights on the dynamic aspects of transcriptional buffering

Next, we focused on transcriptional buffering, a cellular homeostasis mechanism that maintains robust RNA expression levels despite environmental or genetic perturbations in transcription or RNA decay pathways (Timmers and Tora 2018). Transcriptional buffering is often discussed in terms of snapshot changes in nascent RNA levels and RNA half-lives at different steady states, which are typically assessed by metabolic labeling (Rodrigues *et al.* 2023). Accordingly, we aimed to visualize and spotlight on its dynamic aspects, including the processes underlying transcriptional buffering.

**Figure 4.**
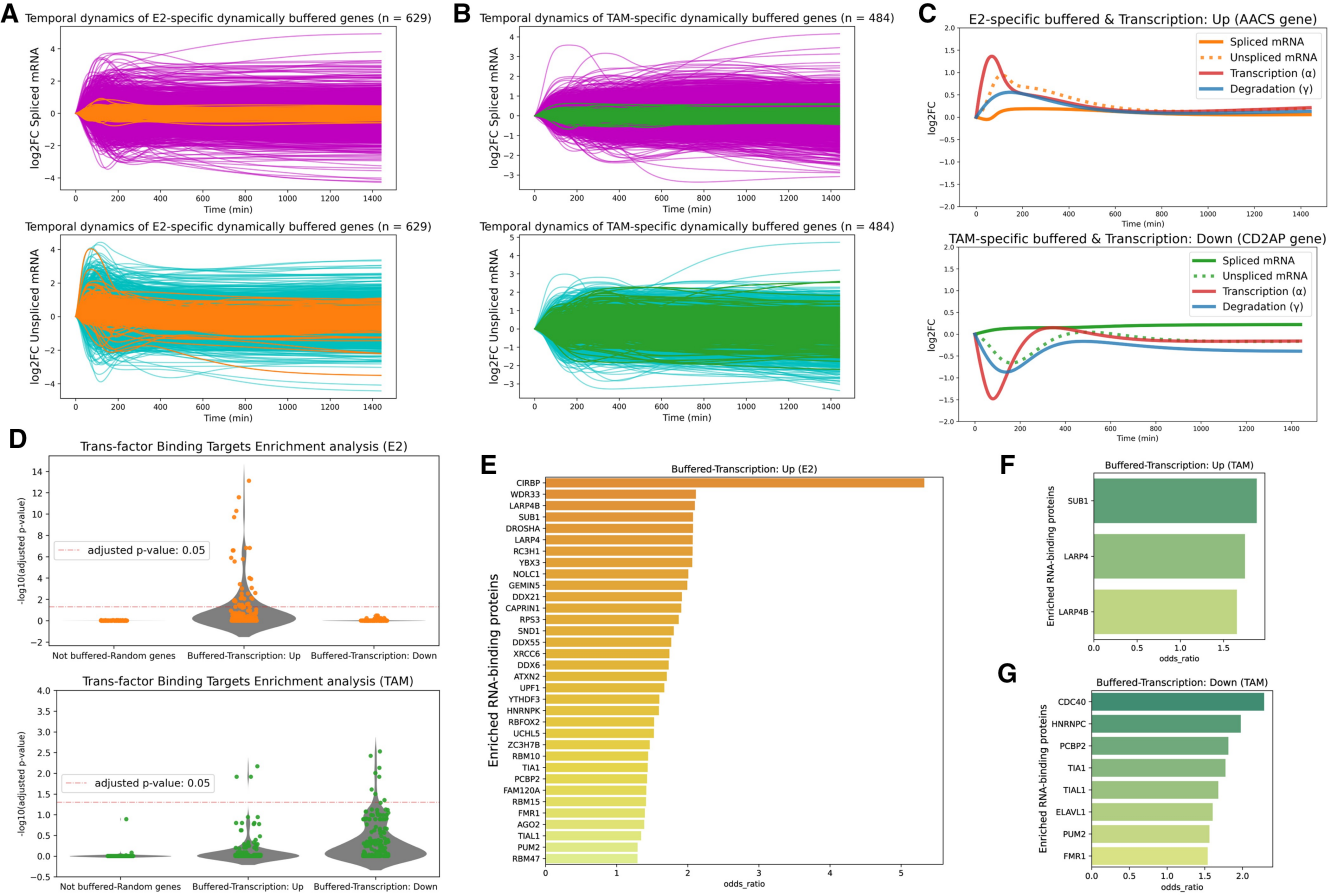
RVINN visualizes the dynamic aspects of transcriptional buffering. (A, B) Temporal dynamics of spliced and unspliced mRNA levels (log2 Fold Change) for E2-specific (A) and TAM-specific (B) dynamically buffered genes. The upper and lower panels show the dynamics of spliced and unspliced mRNA, respectively. Each orange line represents E2-specific dynamically buffered genes, while each green line represents TAM-specific dynamically buffered genes. Other genes are represented in magenta and cyan. (C) Representative examples of E2-specific (AACS gene) and TAM-specific (CD2AP gene) dynamically buffered genes, showing temporal patterns where both transcription and degradation rates are upregulated (upper panel) and where both are downregulated (lower panel). (D) Enrichment analysis of RNA-binding protein (RBP) target genes for E2-specific and TAM-specific dynamically buffered genes. Buffered genes are categorized into “Up” and “Down” groups based on their transcription rate. We performed two-sided Fisher’s exact test to assess whether the known target genes of each expressed RBP are significantly enriched or depleted in each gene group. The viloin plot shows each RBP-target enrichment test with their adjusted *P*-values corrected by the BH method. The horizontal lines indicate the threshold for significance at an adjusted *P*-value of .05. For comparison, the same analysis was performed on random subsets of the non-buffered genes, sampled to match the number of E2-specific or TAM-specific dynamically buffered genes (“Not buffered-random genes” group). (E) Bar plot displaying each RBP whose target genes are significantly enriched in E2-specific dynamically buffered “Transcription Up” genes, ranked by odds ratio. (F, G) Similar to (E), these plots apply to TAM-specific dynamically buffered genes and are visualized for “Transcription Up” (F) and “Transcription Down” (G) cases.

At first, we defined “dynamically buffered” genes as those with relatively smaller standard deviations in spliced mRNA dynamics compared to unspliced mRNA dynamics, and that also have a cross-correlation coefficient above 0.75 between transcription and degradation dynamics within a 60-min time lag (Supplementary Fig. S6A). We used a 60-min time lag for transcription and degradation adjustments based on previous studies indicating that transcriptional buffering may take approximately 60 min (Timmers and Tora 2018). To focus on perturbation-dependent dynamic buffering, we analysed E2-specific dynamically buffered genes (629 genes) and TAM-specific dynamically buffered genes (484 genes) (Supplementary Fig. S6B). We visualized the time-course profiles of these perturbation-specific dynamically buffered genes (Fig. 4A and B), suggesting that stable spliced mRNA expression patterns observed under perturbation are maintained through the dynamic cooperation of transcriptional and post-transcriptional regulation (Fig. 4C). Previous studies have emphasized the involvement of RNA-binding proteins (RBPs) in transcriptional buffering, suggesting that different RBPs may be involved in buffering depending on whether the transcription rate increases or decreases (Rodrigues *et al.* 2023). In particular, specific RBPs involved in transcriptional buffering have been reported as potential therapeutic targets (Todorovski *et al.* 2024). Therefore, we investigated whether there were differences in putative molecular mechanisms depending on their buffering direction by categorizing genes into two subgroups based on the maximum log2-transformed fold change in transcription rate within the first 60 min: those with a positive change (Transcription Up genes) and those with a negative change (Transcription Down genes). The E2-specific buffered 629 genes were categorized into 527 genes in the Transcription Up subgroup and 102 genes in the Transcription Down subgroup, while the TAM-specific buffered 484 genes were categorized into 240 genes in the Transcription Up subgroup and 244 genes in the Transcription Down subgroup.

Using RBP-target data from AURA2, a database of experimentally validated RBPs and their target genes (Dassi *et al.* 2014), we performed an enrichment analysis to test the over-representation of RBP-target genes within each buffered-gene subgroup for each expressed RBP in MCF-7 cells (Fig. 4D). Notably, among the E2-specific dynamically buffered genes, only the Transcription Up subgroup showed significant enrichment of RBP targets (Fig. 4E). A total of 34 RBPs were significant and ranked by their odds ratios, and the top-ranked CIRBP is known to be associated with breast cancer prognosis (Indacochea *et al.* 2021). Additionally, the Transcription Up subgroups of both E2- and TAM-specific dynamically buffered genes commonly include factors such as the LARP gene family, which have been reported to be associated with transcriptional buffering through nucleocytoplasmic shuttling (Gilbertson *et al.* 2018) (Fig. 4F). In contrast, the Transcription Down subgroup of TAM-specific dynamically buffered genes includes factors such as TIA1 and TIAL1, which are components of stress granules (Kedersha *et al.* 1999), as well as ELAVL1 which is involved in RNA stability (Todorovski *et al.* 2024) (Fig. 4G).

These results suggest that RVINN enables to capture the dynamic aspects of transcriptional buffering and provides valuable insights into its molecular mechanisms.

## 4 Discussion

In this study, we developed RVINN which is a flexible modeling for inferring transcriptional and post-transcriptional dynamics, and validated it using both simulated and experimental data. RVINN leverages neural networks and automatic differentiation to represent dynamic systems based on the ODE model, allowing smooth and flexible representations with the straightforward integration of prior knowledge. Furthermore, it is tackling challenges related to genome-wide measurements, temporal resolution, and experimental artifacts that have constrained earlier methods using RNA probes or metabolic labeling. We believe that it shows promise for applications in various biological phenomena and experimental designs, such as drug responses in cancer cells. As use cases in this study, we demonstrated the ability to computationally revalidate and capture dynamic biological phenomena, such as transcriptional ripple, co-bursting, and buffering. Additionally, our results highlight how the estimated kinetic parameters are linked to molecular mechanisms, such as enhancers and RBPs. Although our work does not aim to claim superiority over studies based on specialized experimental measurements, we propose RVINN as a hypothesis-generating and screening tool that complements these research areas and may evolve along with future measurement technologies.

One potential limitation of this study is the possible overestimation of pre-mRNA abundance in genes with intron retention. This is a challenge not only for our method but also for all existing approaches that use intronic reads as a proxy for pre-mRNA abundance. In particular, it is technically difficult to accurately identify and quantify intron retention using conventional short-read data, as this may introduce an additional layer of bias on data preprocessing (David *et al.* 2022). To mitigate this issue, future studies could incorporate long-read sequencing data or utilize samples separated by nuclear and cytoplasmic fractionation to more accurately resolve splicing states. Additionally, analysing how our model performs on genes with experimentally validated intron retention could provide valuable insights for refinements.

Finally, we discuss potential future directions for extending the current modeling framework. Taking the advantages of a differentiable model, we plan to use the time derivatives of the model-estimated parameters to model gene regulatory networks. Specifically, we consider integrating other omics data and RVINN to facilitate a more comprehensive description of the regulatory factor interactions. In addition to the above, it is also possible to combine Neural ODE-based methods with RVINN. This integration may help reduce challenges such as the estimation of initial conditions and long computation time in the Neural ODE framework (Chen *et al.* 2018a). RVINN has extensibility to various differential equation-based models and can easily incorporate domain-specific knowledge, and we plan to further develop it by leveraging its noted advantages.

In conclusion, we present a novel approach for inferring transcriptional and post-transcriptional regulation, offering valuable insights into experimental and systems biology.

## Supplementary Material

btaf180_Supplementary_Data

## Data Availability

The data underlying this article are available at https://github.com/omuto/RVINN.git or doi: 10.5281/zenodo.15281952.
