# Supplementary material for: RVINN: a flexible modeling for inferring dynamic transcriptional and post-transcriptional regulation using physics-informed neural networks
Source: Bioinformatics. 2025 Jul 15;41(Suppl 1):i561–70. doi: 10.1093/bioinformatics/btaf180 (PMC12261459; doi:10.1093/bioinformatics/btaf180)
Supplement: btaf180_Supplementary_Data [file btaf180_supplementary_data.pdf]

# Supplementary materials

**Muto, et al. "RVINN: A Flexible Modeling for Inferring Dynamic Transcriptional and Post-Transcriptional Regulation Using Physics-Informed Neural Networks"**

## 1. SIMULATION DATA GENERATION

### A. The steady-to-steady scenario

In the steady-to-steady scenario, we generated the transcription $\alpha$ and degradation $\gamma$ rates that mimic the dynamic responses after perturbations. (1) We utilized `scipy.signal` Python package and first initialized second-order transfer functions (`signal.TransferFunction`) with randomized natural frequencies and damping ratios, and generated impulse or step responses (`signal.impulse` or `signal.step`) which were randomly assigned to transcription $\alpha$ and degradation $\gamma$ rate dynamics. Simultaneously, splicing rates $\beta$ (constants) were randomly generated. (2) We simulated the mRNA dynamics using the sets of kinetic parameters and an ODE solver (`scipy.integrate.odeint`).

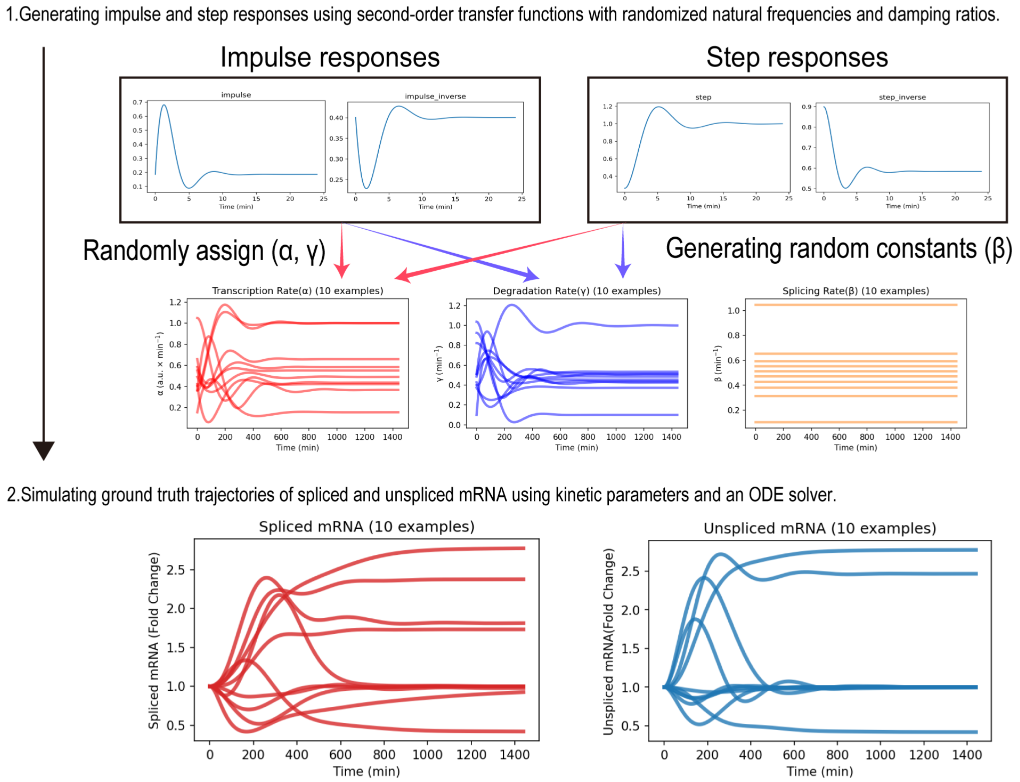


**Fig. S1.** Simulation workflow in the steady-to-steady scenario.

## B. The oscillating scenario

In the oscillating scenario, we used custom oscillating functions that combine exponential and sine wave functions for the transcription $\alpha$ and degradation $\gamma$ rates to generate oscillating gene expression patterns. (1) The amplitude, frequency, and phase of the sine wave function were randomly initialized, and they were substituted into the exponential function. These custom oscillating functions were then randomly assigned to $\alpha$ and $\gamma$. Simultaneously, constants $\beta$ were randomly generated. (2) We simulated the mRNA dynamics using the sets of kinetic parameters and an ODE solver (`scipy.integrate.odeint`).

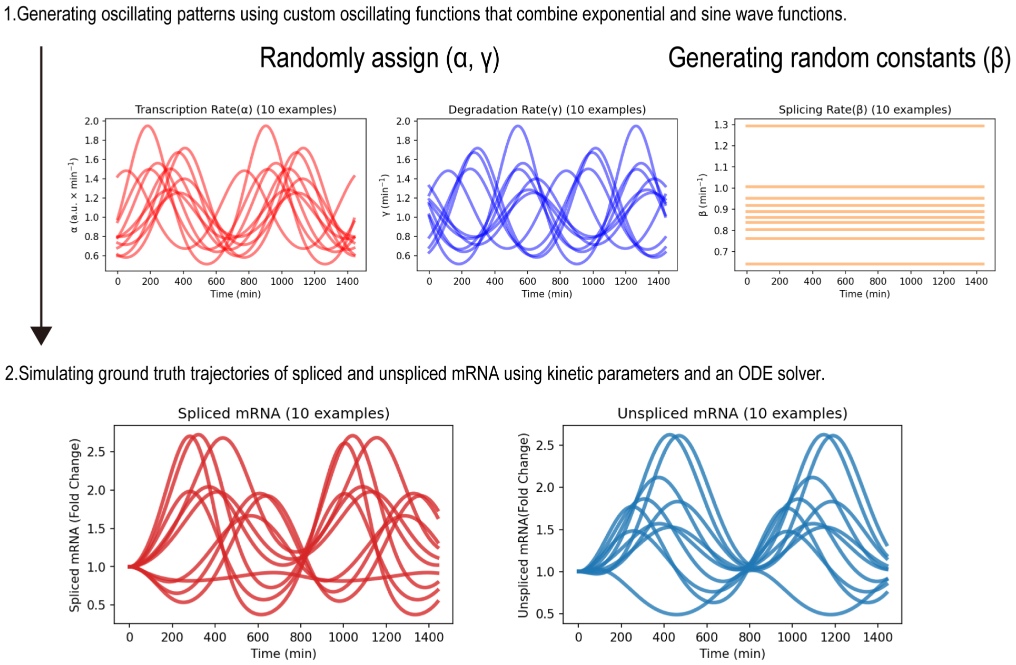


**Fig. S2.** Simulation workflow in the oscillating scenario.

## 2. SIMULATION-BASED EVALUATION OF RVINN

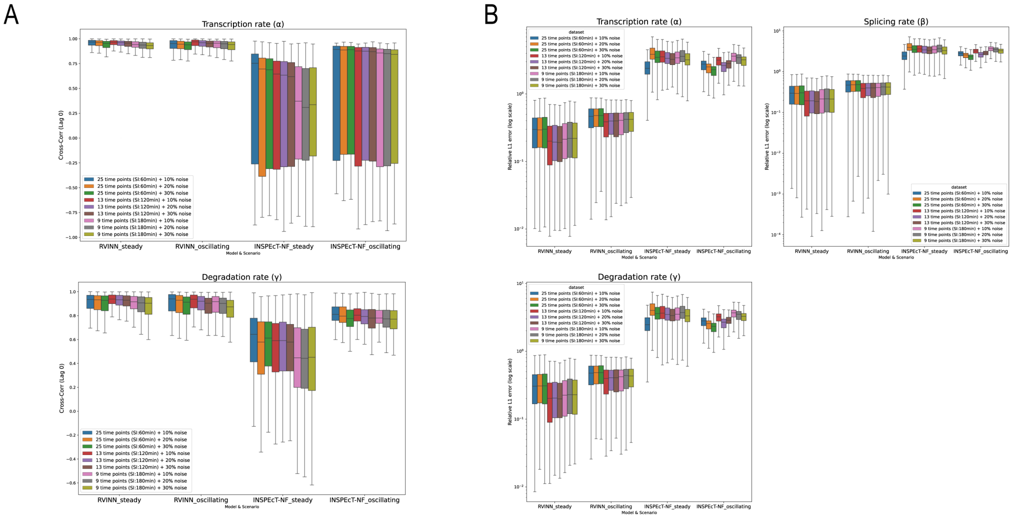


**Fig. S3.** Performance evaluation of RVINN in estimating kinetic parameters for simulated datasets. (A) Box plots show the cross-correlation coefficients (lag 0) between the estimated and ground-truth transcription rate ($\alpha$) and degradation rate ($\gamma$) using RVINN or INSPEcT (NF mode). The x-axis indicates the methods and scenarios (steady-to-steady or oscillating), and the y-axis shows the cross-correlation coefficients. Different box plot colors correspond to different experimental setups (number of time points, noise levels). Note that the cross-correlation is undefined for the splicing rate ($\beta$), since its ground-truth values were time-invariant. (B) Box plots show the values of relative $L_1$-error between the estimated and ground-truth values for $\alpha$, $\beta$, and $\gamma$. The y-axis is plotted on a log10 scale, and the dataset setups are consistent with panel (A).

| Scenario | Dataset | Method | Transcription ($\alpha$) | | Splicing ($\beta$) | Degradation ($\gamma$) | |
|---|---|---|---|---|---|---|---|
| | | | Cross-Corr | Rel L1-error | Rel L1-error | Cross-Corr | Rel L1-error |
| steady | 25 time points + 10% noise | RVINN | 0.97(0.05) | 3.0e-01(2.8e-01) | 3.0e-01(2.8e-01) | 0.94(0.11) | 3.1e-01(2.8e-01) |
| steady | 25 time points + 20% noise | RVINN | 0.96(0.05) | 2.9e-01(2.8e-01) | 2.9e-01(2.8e-01) | 0.93(0.12) | 3.1e-01(2.8e-01) |
| steady | 25 time points + 30% noise | RVINN | 0.95(0.06) | 3.0e-01(2.9e-01) | 3.0e-01(3.0e-01) | 0.93(0.13) | 3.1e-01(3.0e-01) |
| steady | 13 time points + 10% noise | RVINN | 0.97(0.04) | 2.0e-01(2.5e-01) | 1.9e-01(2.5e-01) | 0.94(0.07) | 2.0e-01(2.5e-01) |
| steady | 13 time points + 20% noise | RVINN | 0.96(0.05) | 1.9e-01(2.4e-01) | 1.9e-01(2.4e-01) | 0.93(0.08) | 2.0e-01(2.4e-01) |
| steady | 13 time points + 30% noise | RVINN | 0.96(0.05) | 1.9e-01(2.3e-01) | 1.9e-01(2.4e-01) | 0.93(0.09) | 2.0e-01(2.3e-01) |
| steady | 9 time points + 10% noise | RVINN | 0.94(0.06) | 2.1e-01(2.5e-01) | 2.1e-01(2.7e-01) | 0.92(0.10) | 2.3e-01(2.6e-01) |
| steady | 9 time points + 20% noise | RVINN | 0.94(0.06) | 2.2e-01(2.7e-01) | 2.2e-01(2.8e-01) | 0.91(0.13) | 2.3e-01(2.7e-01) |
| steady | 9 time points + 30% noise | RVINN | 0.93(0.06) | 2.2e-01(2.6e-01) | 2.1e-01(2.7e-01) | 0.90(0.14) | 2.3e-01(2.6e-01) |
| steady | 25 time points + 10% noise | INSPEcT-NF | 0.75(1.11) | 2.4e+00(1.0e+00) | 2.4e+00(1.1e+00) | 0.64(0.37) | 2.5e+00(1.1e+00) |
| steady | 25 time points + 20% noise | INSPEcT-NF | 0.70(1.19) | 3.9e+00(1.5e+00) | 3.9e+00(1.6e+00) | 0.58(0.44) | 4.0e+00(1.6e+00) |
| steady | 25 time points + 30% noise | INSPEcT-NF | 0.69(1.11) | 3.5e+00(1.4e+00) | 3.5e+00(1.4e+00) | 0.61(0.37) | 3.6e+00(1.5e+00) |
| steady | 13 time points + 10% noise | INSPEcT-NF | 0.64(1.09) | 3.5e+00(1.4e+00) | 3.6e+00(1.4e+00) | 0.59(0.41) | 3.6e+00(1.6e+00) |
| steady | 13 time points + 20% noise | INSPEcT-NF | 0.63(1.06) | 3.4e+00(1.3e+00) | 3.4e+00(1.4e+00) | 0.59(0.41) | 3.5e+00(1.5e+00) |
| steady | 13 time points + 30% noise | INSPEcT-NF | 0.62(1.05) | 3.3e+00(1.3e+00) | 3.3e+00(1.3e+00) | 0.58(0.39) | 3.4e+00(1.4e+00) |
| steady | 9 time points + 10% noise | INSPEcT-NF | 0.37(0.93) | 3.4e+00(1.4e+00) | 3.5e+00(1.4e+00) | 0.45(0.50) | 3.5e+00(1.5e+00) |
| steady | 9 time points + 20% noise | INSPEcT-NF | 0.31(0.92) | 3.6e+00(1.4e+00) | 3.7e+00(1.5e+00) | 0.44(0.49) | 3.7e+00(1.6e+00) |
| steady | 9 time points + 30% noise | INSPEcT-NF | 0.34(0.89) | 3.2e+00(1.3e+00) | 3.3e+00(1.4e+00) | 0.45(0.53) | 3.3e+00(1.4e+00) |
| oscillating | 25 time points + 10% noise | RVINN | 0.95(0.08) | 4.7e-01(2.9e-01) | 4.7e-01(2.9e-01) | 0.94(0.14) | 4.8e-01(2.9e-01) |
| oscillating | 25 time points + 20% noise | RVINN | 0.95(0.08) | 4.8e-01(2.7e-01) | 4.8e-01(2.7e-01) | 0.93(0.14) | 4.8e-01(2.8e-01) |
| oscillating | 25 time points + 30% noise | RVINN | 0.94(0.08) | 4.8e-01(2.8e-01) | 4.8e-01(2.8e-01) | 0.91(0.15) | 4.8e-01(2.8e-01) |
| oscillating | 13 time points + 10% noise | RVINN | 0.97(0.06) | 3.9e-01(2.9e-01) | 3.9e-01(2.9e-01) | 0.94(0.11) | 4.0e-01(3.0e-01) |
| oscillating | 13 time points + 20% noise | RVINN | 0.96(0.06) | 4.0e-01(2.7e-01) | 4.0e-01(2.6e-01) | 0.92(0.11) | 4.1e-01(2.6e-01) |
| oscillating | 13 time points + 30% noise | RVINN | 0.95(0.06) | 4.0e-01(2.9e-01) | 4.0e-01(3.0e-01) | 0.90(0.13) | 4.1e-01(2.8e-01) |
| oscillating | 9 time points + 10% noise | RVINN | 0.96(0.07) | 4.1e-01(2.7e-01) | 4.1e-01(2.7e-01) | 0.92(0.13) | 4.2e-01(2.7e-01) |
| oscillating | 9 time points + 20% noise | RVINN | 0.95(0.07) | 4.2e-01(2.6e-01) | 4.2e-01(2.6e-01) | 0.90(0.13) | 4.4e-01(2.6e-01) |
| oscillating | 9 time points + 30% noise | RVINN | 0.94(0.08) | 4.2e-01(2.5e-01) | 4.2e-01(2.6e-01) | 0.87(0.14) | 4.3e-01(2.5e-01) |
| oscillating | 25 time points + 10% noise | INSPEcT-NF | 0.89(1.15) | 2.8e+00(8.0e-01) | 2.8e+00(6.7e-01) | 0.81(0.11) | 2.8e+00(7.2e-01) |
| oscillating | 25 time points + 20% noise | INSPEcT-NF | 0.89(1.09) | 2.5e+00(7.4e-01) | 2.6e+00(6.4e-01) | 0.80(0.13) | 2.6e+00(6.8e-01) |
| oscillating | 25 time points + 30% noise | INSPEcT-NF | 0.89(1.09) | 2.3e+00(6.7e-01) | 2.3e+00(6.0e-01) | 0.78(0.14) | 2.3e+00(6.5e-01) |
| oscillating | 13 time points + 10% noise | INSPEcT-NF | 0.88(1.20) | 3.2e+00(8.4e-01) | 3.2e+00(7.1e-01) | 0.80(0.10) | 3.2e+00(7.7e-01) |
| oscillating | 13 time points + 20% noise | INSPEcT-NF | 0.88(1.14) | 2.6e+00(7.9e-01) | 2.7e+00(6.9e-01) | 0.79(0.12) | 2.7e+00(7.7e-01) |
| oscillating | 13 time points + 30% noise | INSPEcT-NF | 0.87(1.14) | 2.8e+00(7.2e-01) | 2.9e+00(6.0e-01) | 0.78(0.16) | 2.9e+00(6.0e-01) |
| oscillating | 9 time points + 10% noise | INSPEcT-NF | 0.86(1.19) | 3.6e+00(1.0e+00) | 3.7e+00(8.1e-01) | 0.78(0.11) | 3.7e+00(8.5e-01) |
| oscillating | 9 time points + 20% noise | INSPEcT-NF | 0.85(1.17) | 3.4e+00(1.0e+00) | 3.5e+00(8.3e-01) | 0.77(0.14) | 3.5e+00(9.3e-01) |
| oscillating | 9 time points + 30% noise | INSPEcT-NF | 0.84(1.15) | 3.2e+00(9.1e-01) | 3.3e+00(7.4e-01) | 0.77(0.15) | 3.3e+00(7.7e-01) |

**Table S1.** Table of evaluation metrics on RVINN and INSPEcT (NF mode) in simulated datasets across steady-to-steady and oscillating scenarios. Each value represents the median with the interquartile range (IQR) of cross-correlation coefficients (Cross-Corr) and relative L1-error (Rel L1-error) for the kinetic parameters.

| Scenario | Dataset | Method | Transcription ($\alpha$) | | Splicing ($\beta$) | Degradation ($\gamma$) | |
|---|---|---|---|---|---|---|---|
| | | | Cross-Corr | Rel L1-error | Rel L1-error | Cross-Corr | Rel L1-error |
| steady | 25 time points + 10% noise | RVINN | 0.98(0.04) | 2.5e-01(2.7e-01) | 2.5e-01(2.8e-01) | 0.94(0.09) | 2.6e-01(2.8e-01) |
| steady | 25 time points + 20% noise | RVINN | 0.97(0.04) | 2.4e-01(2.6e-01) | 2.4e-01(2.7e-01) | 0.94(0.10) | 2.4e-01(2.8e-01) |
| steady | 25 time points + 30% noise | RVINN | 0.96(0.06) | 2.7e-01(2.7e-01) | 2.6e-01(2.8e-01) | 0.92(0.13) | 2.8e-01(2.8e-01) |
| steady | 13 time points + 10% noise | RVINN | 0.97(0.04) | 1.9e-01(2.7e-01) | 1.9e-01(2.6e-01) | 0.93(0.08) | 2.0e-01(2.6e-01) |
| steady | 13 time points + 20% noise | RVINN | 0.96(0.05) | 2.1e-01(2.9e-01) | 2.1e-01(2.8e-01) | 0.93(0.09) | 2.2e-01(2.8e-01) |
| steady | 13 time points + 30% noise | RVINN | 0.95(0.05) | 2.5e-01(3.3e-01) | 2.3e-01(3.2e-01) | 0.90(0.15) | 2.4e-01(3.0e-01) |
| steady | 9 time points + 10% noise | RVINN | 0.95(0.06) | 2.3e-01(3.0e-01) | 2.3e-01(3.1e-01) | 0.90(0.12) | 2.4e-01(3.0e-01) |
| steady | 9 time points + 20% noise | RVINN | 0.95(0.06) | 2.6e-01(3.6e-01) | 2.5e-01(3.6e-01) | 0.90(0.14) | 2.6e-01(3.5e-01) |
| steady | 9 time points + 30% noise | RVINN | 0.93(0.07) | 2.6e-01(3.0e-01) | 2.6e-01(3.0e-01) | 0.91(0.15) | 2.8e-01(2.8e-01) |
| steady | 25 time points + 10% noise | INSPEcT-Default | 0.68(0.24) | 1.2e+01(4.1e+00) | 1.2e+01(4.2e+00) | 0.22(0.91) | 1.2e+01(4.2e+00) |
| steady | 25 time points + 20% noise | INSPEcT-Default | 0.66(0.25) | 1.1e+01(4.5e+00) | 1.1e+01(4.3e+00) | 0.25(0.91) | 1.1e+01(4.3e+00) |
| steady | 25 time points + 30% noise | INSPEcT-Default | 0.65(0.26) | 1.1e+01(5.1e+00) | 1.1e+01(5.0e+00) | 0.32(0.88) | 1.1e+01(4.7e+00) |
| steady | 13 time points + 10% noise | INSPEcT-Default | 0.70(0.21) | 1.0e+01(4.2e+00) | 1.1e+01(4.2e+00) | 0.27(0.94) | 1.1e+01(4.2e+00) |
| steady | 13 time points + 20% noise | INSPEcT-Default | 0.67(0.29) | 1.1e+01(5.1e+00) | 1.1e+01(5.1e+00) | 0.29(0.93) | 1.1e+01(4.9e+00) |
| steady | 13 time points + 30% noise | INSPEcT-Default | 0.67(0.29) | 1.0e+01(5.8e+00) | 1.0e+01(5.4e+00) | 0.35(0.80) | 1.0e+01(5.4e+00) |
| steady | 9 time points + 10% noise | INSPEcT-Default | 0.59(0.42) | 1.1e+01(4.9e+00) | 1.1e+01(4.8e+00) | 0.26(0.88) | 1.1e+01(4.8e+00) |
| steady | 9 time points + 20% noise | INSPEcT-Default | 0.56(0.42) | 1.2e+01(6.5e+00) | 1.2e+01(6.4e+00) | 0.33(0.88) | 1.2e+01(5.9e+00) |
| steady | 9 time points + 30% noise | INSPEcT-Default | 0.57(0.42) | 1.0e+01(5.7e+00) | 1.1e+01(5.8e+00) | 0.33(0.97) | 1.1e+01(5.5e+00) |

**Table S2.** Table of evaluation metrics on RVINN and INSPEcT (default mode) in simulated datasets across steady-to-steady scenarios. Each value represents the median with the interquartile range (IQR) of cross-correlation coefficients (Cross-Corr) and relative L1-error (Rel L1-error) for the kinetic parameters.
Important note: INSPEcT default mode models kinetic parameters using constant, impulse, or sigmoid functions. Therefore, oscillating scenarios were excluded from evaluation. Moreover, genes whose transcription or degradation rates were estimated as constants in INSPEcT default mode were excluded from the analysis, as their correlation coefficients could not be determined.

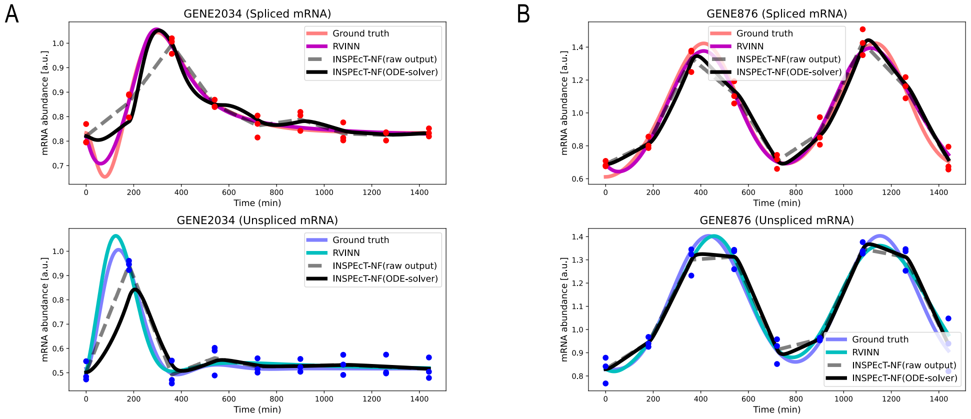


**Fig. S4.** Temporal gene expression profiles for the same genes shown in the main Fig.2, simulated under the steady-to-steady scenario (A) and the oscillating scenario (B). Solid black lines represent solution trajectories obtained by solving the initial value problem using an ODE solver (`scipy.integrate.odeint`) with the initial values and kinetic parameters estimated by INSPEcT (NF mode).

## 3. TRANSCRIPTIONAL RIPPLE

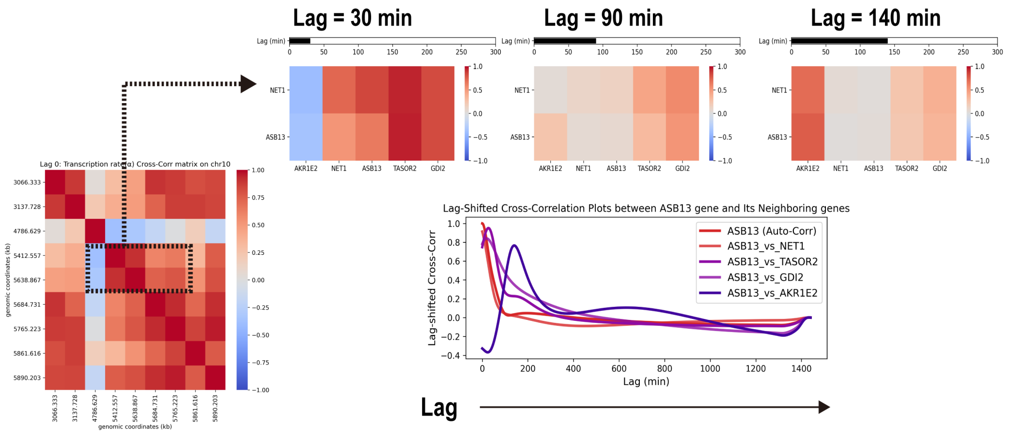


**Fig. S5.** Lag-shifted cross-correlation analysis of ASB13, NET1, and the neighboring genes under E2 treatment. Top panels show lag-shifted cross-correlation matrices at 30, 90, and 140 minutes, showing ripple-like transcriptional activation. The bottom-right panel displays lag-shifted cross-correlation plots between ASB13 and its neighbors.

## 4. DYNAMICALLY BUFFERED GENES

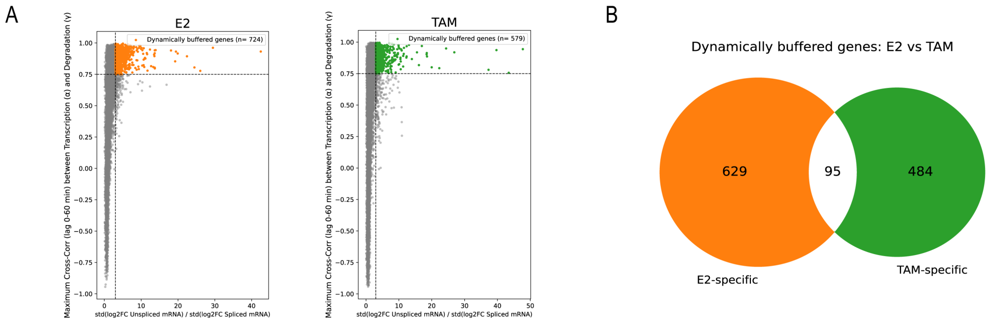


**Fig. S6.** Definition of dynamically buffered genes in MCF-7 cells treated with Estradiol (E2) or Tamoxifen (TAM). (A) The x-axis represents the ratio of the standard deviations of log2-transformed fold-change (FC) of unspliced mRNA dynamics to spliced mRNA dynamics for each gene. The y-axis displays the maximum cross-correlation coefficient between the estimated transcription rate and degradation rate dynamics for each gene, evaluated across time lags (0–60 minutes). Genes with the ratio above 3 (vertical dashed line) and the maximum cross-correlation coefficient above 0.75 (horizontal dashed line) are defined as dynamically buffered genes. Dynamically buffered genes are highlighted in orange (E2-treatment) and green (TAM-treatment), with the number of genes indicated in each category. (B) Venn diagram illustrating the number of E2-specific and TAM-specific dynamically buffered genes.
